# Using species distribution modeling to delineate the botanical richness patterns and phytogeographical regions of China

**DOI:** 10.1038/srep22400

**Published:** 2016-03-01

**Authors:** Ming-Gang Zhang, J. W. Ferry Slik, Ke-Ping Ma

**Affiliations:** 1 State Key Laboratory of Vegetation and Environmental Change, Institute of Botany, Chinese Academy of Sciences, Beijing 100093, China; 2 Institute of Loess Plateau, Shanxi University, Taiyuan 030006, China; 3 Faculty of Science, Universiti Brunei Darussalam, Jln Tungku Link, Gadong BE1410, Brunei Darussalam

## Abstract

The millions of plant specimens that have been collected and stored in Chinese herbaria over the past ~110 years have recently been digitized and geo-referenced. Here we use this unique collection data set for species distribution modeling exercise aiming at mapping & explaining the botanical richness; delineating China’s phytogeographical regions and investigating the environmental drivers of the dissimilarity patterns. We modeled distributions of 6,828 woody plants using MaxEnt and remove the collection bias using null model. The continental China was divided into different phytogeographical regions based on the dissimilarity patterns. An ordination and Getis-Ord Gi* hotspot spatial statistics were used to analysis the environmental drivers of the dissimilarity patterns. We found that the annual precipitation and temperature stability were responsible for observed species diversity. The mechanisms causing dissimilarity pattern seems differ among biogeographical regions. The identified environmental drivers of the dissimilarity patterns for southeast, southwest, northwest and northeast are annual precipitation, topographic & temperature stability, water deficit and temperature instability, respectively. For effective conservation of China’s plant diversity, identifying the historical refuge and protection of high diversity areas in each of the identified floristic regions and their subdivisions will be essential.

In the past ~110 years, millions of specimens have been collected and stored in Chinese herbaria. Over 11,400 woody plants have been identified^1^, with about 7,000 of these (*c.* 61%) endemic^2^. Based on the distribution information of the specimens, efforts have been made to map and explain the geographical variation in plant species richness across China^3^,^4^, identifying both centers of endemicity^2^ and refugia^5^. Explaining the large scale geographical variation in plant species richness is one of the key issues in ecology and the efforts have typically focused on effects of either ‘history’ or ‘ecology’^6^. Some ‘history’ related hypotheses state that tropical areas have higher rates of speciation, and that at higher latitudes most tropical species cannot tolerate the abiotic stress (e.g.: cold temperature, water deficit and extreme seasonality) due to evolutionary constraints on their niche breadth^7^. Other historical hypotheses propose that in habitats with a stable environment species have enough time to disperse and adapt, thus the diversity patterns reflect a steady-state relationship between the environment and the evolutionary processes^8^. Alternatively, the ‘ecology’ related hypotheses initially focus on the productivity of terrestrial environments. The productivity of ecosystems is initially considered to be determined by energy or the combined effects of water availability and environmental energy^9^. The strong environmental gradients, complex topography and a long geological history make China a ‘natural lab’ for testing these hypotheses^10^.

The geographical patterns of life on earth are shaped by past and current physical and biological forces, and can be summarized by biogeographical regionalizations^11^. When exploring basic and applied questions in biogeography, evolutionary biology, systematics, ecology and conservation, these biogeographical regionalizations provide an indispensable background against which to interpret research findings^11^,^12^. In recent years, with the increasing availability of large databases on species distributions, the importance of robust systems for classifying biogeographical patterns has been emphasized^8^,^11^. For China, the most comprehensive biogeographical regionalization studies were conducted by Wu^13^, who divided China into 4 major floristic regions, 7 sub-regions, 27 areas and 49 sub-areas. However, these studies lacked high-resolution data on species-level distributions and the robust statistical basis that is available today. Wu *et al.*’s study^13^ also lacked information on the floristic relationships between the identified regions.

Former macro-scale studies were generally based on the raw survey data, which can give rise to several problems. One of these is the geographical collection bias: Most regions of China have incomplete collection effort^14^, also termed ‘inventory incompleteness’ leading to incomplete information on actual ranges for many species^15^. At the same time, the available collection localities generally do not represent random samples from the available environmental gradients, with most collections coming from sites near roads, in protected areas, or areas of known high plant diversity or special species composition^16^. Furthermore, for many regions in China, the original vegetation has disappeared and been replaced by agricultural crops, planted forests, or urbanization. Current collections from these sites thus do no longer represent the actual potential species composition^17^. Thus, when using large species collection databases, statistical procedures that take these problems into account are critical for obtaining accurate diversity and species distributional maps.

Species distribution modeling is now widely used to determine distribution patterns at large spatial scales^18^. Species distribution models attempt to identify and map the suitable habitat range of species by combining environmental predictors with presence records^19^,^20^. Herbarium collection databases generally provide the primary presence data for such analyses. Currently, most specimens available in Chinese herbaria have been digitalized and are available online^15^. Meanwhile, much progress has been made in species distribution modeling, such as dealing with small sample sizes^21^,^22^, and overcoming collection bias using null model tests^23^,^24^. In this study, we aim to (1) use species distribution modeling to map and explain the spatial distribution of the woody plant diversity in mainland China; (2) use the obtained presence/absence data to delineate the phytogeographical regions of China and their relationships at genus and species level; (3) detect the effect of the uncorrelated environmental predictors on the dissimilarity patterns of species.

## Results

**Figure 1:**
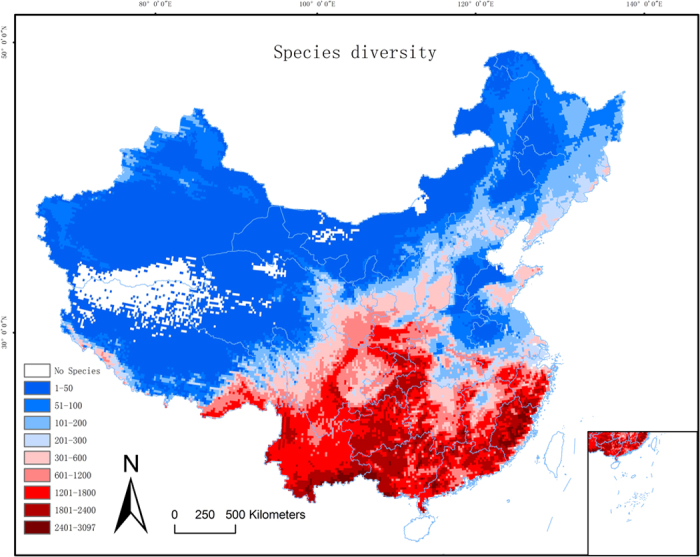
Botanical richness pattern of continent China, 10 × 10 arc-min spatial solution. Albers projection. The map was plotted using ArcGIS 9.3 (http://www.esri.com/).

Of the 34,230 grid cells covering China, 3,068 (9%) have reliably georeferenced plant collections available (Fig. S1). These collecting localities were environmentally biased for 14 of the 15 environmental predictors (Fig. S2). Of the 6,828 modeled species, 6,560 showed significant non-random habitat associations when tested against a collection bias corrected null model (Fig. S3). After stacking of the SDMs of these 6,560 species south-central China showed the highest woody plant diversity (Fig. 1). Mountain ranges in southern China were especially high in diversity, including the Qinling Mountains (28), Daba Mountains (30), Gaoligong Mountains (39), Wuling Mountains (50), Dalou Mountains (52), Daiyun Mountains (56), Nanling Mountains (61), Dayao Mountains (62), and Yunwu Mountains (63). In variation partitioning analysis (Fig. S5), the environmental predictors explained 11% of the species richness independently, while the explanatory power of environmental predictors and spatial predictors combined was 69%. Due to the weak explanatory power (1%) of spatial predictors alone, we only used the environmental predictors to explain the species richness patterns. Among the best predictors of species richness in China were: Annual rainfall (positive), Temperature annual range (negative), Cation-Exchange-Capacity of the clay subsoil (negative), and the topsoil C:N-ratio (positive), with the most important environmental variable (highest β-value) being temperature annual range (Table 1).

**Figure 2:**
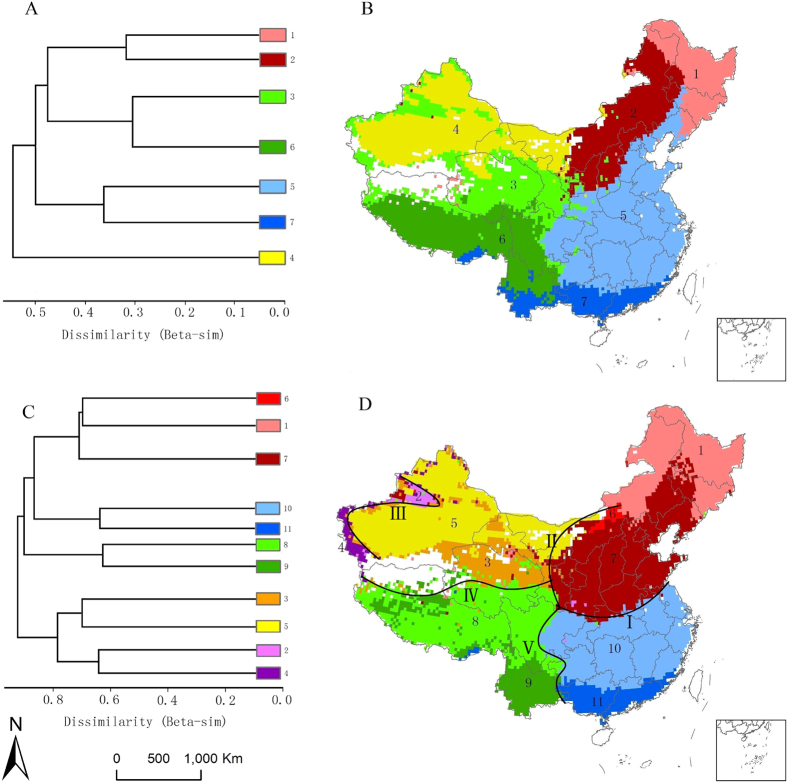
Dendrograms (**A,C**) and maps (**B,D**) resulting from UPGMA hierarchical clustering of grid cell assemblages based on beta-sim dissimilarity matrices for woody plants at the level of genus (**A,B**) and species (**C,D**). Line: I, II, III, IV, V indicate 5 major divisions of continental China. Map B and D are in Albers projection. The map was plotted using ArcGIS 9.3 (http://www.esri.com/).

**Figure 3:**
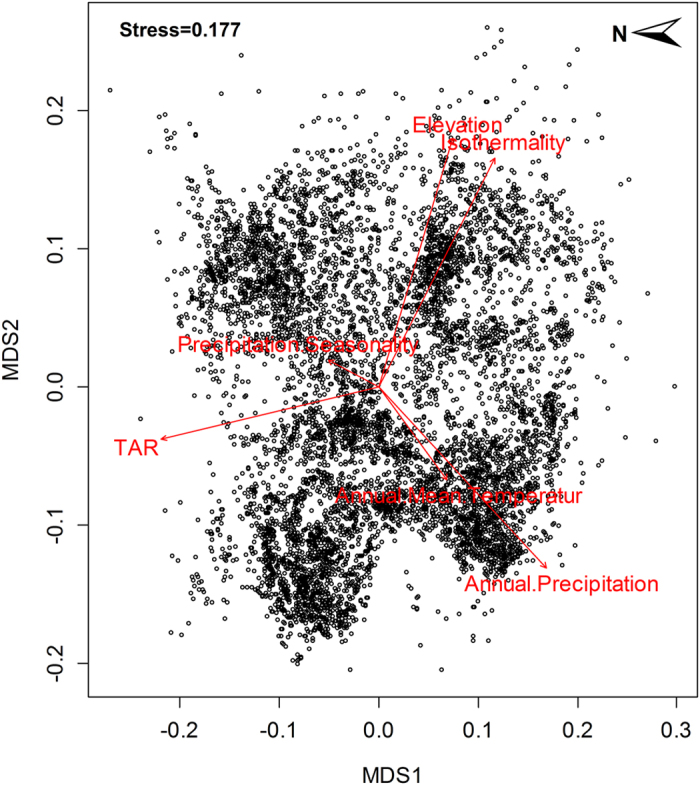
Fifteen uncorrelated variables plotted as predictors of environmental turnover calculated for 6560 woody species. Vectors are displayed only for the highly significant variables (P < 0.001) inferred from non-metric multidimensional scaling (NMDS) ordination. TAR: Temperature Annual Rang.

With cluster analysis at genus level, seven phytogeographical regions belonging to four major groupings were identified in continental China (Fig. 2). At species level eleven phytogeographical regions were identified belonging to five major groupings, and the major groups were segmented by five dividing lines (I, II, III, IV, V in Fig. 2). The order of the major groupings differed between the genus and species level analyses (Fig. 2). In the relative environmental turnover analysis, the stress value (0.177) suggests an acceptable fit of the environmental data to the 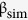 clusters in the NMDS ordination (Fig. 3). At continental level, the species turnover towards northeast China correlated highly with temperature annual range and soil drainage class. The species turnover towards northwest China correlated with precipitation seasonality. The species turnover towards southwest China was highly correlated with elevation range and isothermality. The species turnover towards southeast China strongly correlated with annual precipitation (Fig. 3). The Gi* statistic showed that temperature in northeast China is highly unstable (lowest Isothermality and highest temperature annual range), while the temperature in southwest China is most stable. Northwest China is limited by low temperatures and moisture (lowest annual mean temperature and annual precipitation). Compared with other regions, southeast China has the best moisture conditions (highest annual precipitation) (Table 2).

## Discussion

### Botanical richness patterns and their response to the environment

As a country famous for its extremely high species diversity, mapping the geographic patterns of species richness across China, and detecting the relationship between species richness and environments is critical to help conserve the biodiversity across the country. Former studies highlight the effect of mean temperature of the coldest quarter^10^ and temperature seasonality in shaping the current species diversity patterns in China. Based on the 6,560 significantly nonrandom distributed SDMs, our results suggest the most important variable is annual precipitation. This variable can explain most of richness pattern when tested alone. Meanwhile, their positive correlation suggests that highest richness values are found under relatively high and stable moisture conditions. Temperature annual range explained most of the variance in species richness; their negative correlation suggests that temperature stability is the driving factor for the observed patterns in species richness. Apart from this, the richness pattern showed a limited correlation with soil factors, similar with the former research for woody species in Europe^25^. However, a weak negative correlation between species diversity and soil fertility can still be found in our study.

Water availability is considered as a strong promoter of species richness by promoting ecosystem productivity^26^,^27^. Normally, it is used to explain the diversity pattern associated with energy^28^ and their relative importance was thought to be regional specificity^29^. Water availability was thought to be more important when there is no limitation of energy input^30^. In southern Africa, O’Brien, *et al.*^31^ found that the woody plant diversity was most strongly correlated with water availability. In lowland and montane Neotropical forests, water availability was identified as the dominant predictor of liana species richness and wetter forests had a greater species richness of all woody plants^32^. At global scale, Kreft, *et al.*^33^ found that the water availability is a much stronger constraint of pteridophyte and seed plant richness and humid tropical mountainous regions are conserving most of the pteridophyte richness.

The role of temperature stability in shaping the plant richness pattern has been found by several studies in different continents. In Borneo, the highest species diversity values of woody plants were found under relative stable annual temperature conditions^34^. In South Africa, the high speciation and low extinction rate were proved to be promoted by temperature stability^35^. In Australia, the regions with highest species diversity and endemism were also proved have the highest environmental stability^36^. Recently, many studies even noticed that not only the contemporary climatic stability but the historical climatic stability perform well in predicting the species diversity patterns^37^. For the Amazon, regional diversity is strongly correlated with historical climatic stability^38^, while climatic instability is thought to increase extinction rates^39^,^40^. The strong association between species richness and glacial-interglacial climate stability were also found for plants in Europe^41^. For palm in tropical rain forests, recent research found that historical climatic stability is the main driver for regional species pool, thus promote the local species richness^42^. This finding could be supported by the historical refuga hypotheses in macro-ecology, but the mechanism still need further test in future studies.

### Phytogeographical regions of China and their linkage to the environment

The most comprehensive study on biogeographical regionalization of Chinese plants was carried out by Wu *et al.* (2010). Our 11 biogeographical sub-regions are in broad agreement with Wu’s definition based on qualitative evidence by experts. However, some differences can still be found between our analysis and Wu’s division: (i) Wu’s Paleotropical zone was divided into two parts by a major split (Line V in Fig. 2D) and being separated into Tibetan Plateau region and Southeast China. Meanwhile, a recent study redefined a larger Paleotropical zone based on the genus distribution and suggested that the line at c. 22°30′N is the reasonable northern biogeographical boundary of the tropical zone^43^, which can roughly match our division at genus level; (ii) A sharp change on species composition happened in the middle of Wu’s Sino-Japanese floristic region. The major split (Line I in Fig. 2D) strongly match the 0 °C isotherm in January and it is the traditional division of subtropical and warm temperate zones of China; (iii) No clear Eurasian Steppe region can be identified based on the distribution of woody species; this is no surprise that most woody species are coming from the nearby forest. But the genus composition is still quite different from other areas; (iv) The Tianshan and West Kunlun mountain area is identified as a major biogeographical region instead of being separated into Tibetan Plateau region and Central Asian desert region. Additionally, all of the 11 biogeographical sub-regions can be grouped into 5 major biogeographical regions, except the Tianshan and West-Kunlun mountain regions, other four major regions are in agreement with another comprehensive study on biogeographical regionalization that based on the distribution of 509 higher plants^44^.

The NMDS analysis was an efficient tool in identifying major biogeographical splits in continental China, but less sensitive in identifying the sub-regions^11^. Meanwhile, our analyses confirm the hypothesis that the patterns of species turnover are promoted by the changes in environmental conditions^12^ and the mechanisms causing dissimilarity pattern may differ among biogeographical regions^45^. Southeast China is a typical Monsoon tropical & subtropical region. This is a region with the best moisture conditions and weak limitation from energy input. The precipitation has the gradient from southeast coast to inland, which can be mirrored in the species dissimilarity pattern. This finding can be well supported in a study based on the plots survey in montane forests of China^45^. The Southwest China has a distinct isolation from the Southeast Monsoon area due to the geographical barriers formed by the sharp elevating altitude from east to west. The most special character of this region is that the altitude elevating from the south to the north. Meanwhile, due to lower latitudes and higher elevations, the whole regions shown the most stable temperature conditions, which also varies with the altitude gradient together with the dissimilarity pattern of woody plants. Moving to the northern China, the environmental factors with the largest variation is the temperature annual range. The whole region is suffering from the limited energy input and the dissimilarity pattern of woody plants changes with the increased instability of temperature from south to north. Northwest China is strongly influenced by water input, and the landscape and species composition of this region show great difference with other part of China. Rather than total rainfall, our analysis showed that the seasonal variation of rainfall is the main driver for the dissimilarity pattern. This result accordance with a recent study conducted in the central deserts in Australia^12^.

## Conclusions

For China, the water availability and stability of temperature were identified as the main divers of the contemporary species diversity pattern. The two variables explaining most of the variance of the species richness were comparable to the macro-ecological diversity studies in other regions. Based on the high resolution of species level data and the robust method for classifying biogeographical regions, eleven biogeographical regions and five major divisions were successfully recognized. Different driving forces for the dissimilarity pattern of plants for the five major divisions were identified: the precipitation in southeast China; the elevating of altitude and stable temperature in southwest China; the instability of temperature in northeast China and the water deficit in northwest China. Our study found that for effective conservation of China’s plant diversity, the areas with adequate precipitation and most stable temperature need more attention; the importance of historical refuga needs further test. More importantly, the high diversity areas in each of the identified floristic regions and their subdivisions should be considered with special priority for plant conservation.

## Methods

### Species data

We obtained collection information for *c.* 4.5 million specimens of Chinese vascular plants present in 42 major herbaria from the Chinese Virtual Herbarium (http://www.cvh.org.cn/cms/en/, accessed September 2012). After removing all non-woody species, 1.1 million specimens (trees, shrubs and lianas) remained. However, most of these had no latitude and longitude information. These collections were georeferenced using the location descriptions as provided on the labels. This resulted in 464,045 specimens with accurate location data. Subsequently, species presences were scored in 10 arc-minute grid cells, avoiding duplicate species records in each grid cell. We used the 10 arc-minute spatial resolutions because this corresponded to the environmental data resolution (WorldClim), but more importantly, because a higher resolution was not possible due to the spatial error in the georeferenced specimen data. Species that were present in fewer than 5 grid cells were removed from the analysis. Of the 464,045 georeferenced specimens, 371,712 records belonging to 157 plant families representing 6,828 species were finally kept to be modeled.

### Environmental predictors

Initially, 35 environmental predictors were selected to model the species distribution patterns. These included 19 bioclimatic predictors (1950–2000) plus altitude of the WORLDCLIM dataset (www.worldclim.org) for China at 10 arc-minute resolution and 15 soil variables selected from the FAO database for poverty and insecurity mapping^46^. The FAO soil properties had a spatial resolution of 5 arc-minute, so we re-sampled all soil layers into 10 arc-minute grid cells. The whole mainland of China was covered by 34,230 grid cells with a resolution of 10-arc minutes.

Because multi-colinearity of variables can result in over-fitting in species distribution modeling^22^,^47^, we removed highly correlated environmental predictors. For both bio-climatic and soil predictors, we used spearman’s rank correlation tests to select the least correlated variables (spearman’s rho < 0.75). From correlated variables with Spearman rho higher than 0.75 only the ecologically most meaningful factors were kept. This procedure eventually resulted in the following climatic variables being included in further analyses: (1) Bio01: Annual Mean Temperature; (2) Bio03: Isothermality (P2/P7) × 100 (P2: Mean Diurnal Rang; P7: Temperature Annual Range); (3) Bio07: Temperature Annual Range; (4) Bio12: Annual Precipitation; (5) Bio15: Precipitation Seasonality (Table S1). Of the soil predictors the following variables were included in the analysis: (1) BS-T: base saturation% topsoil; (2) CE-S: CEC clay subsoil (CEC = cation exchange capacity); (3) CN-T: Carbon (C) : Nitrogen (N) ratio class topsoil; (4) CP-T: organic carbon pool topsoil; (5) Depth: effective soil depth; (6) Drain: soil drainage class; (7) NN-T: nitrogen% topsoil; (8) Prod: soil production index; (9) Text.: textural class subsoil (Table S2). In total, 15 of the original 35 predictors were kept to model the species distributions.

### Species distribution model building and collection bias correction

In order to model species distributions we used the modeling application Maxent (ver. 3.3.3k; www.cs.princeton.edu/~schapire/maxent/)^20^. Maxent was specifically developed to model species distributions with presence-only data. Of available species distribution modeling algorithms, Maxent has been shown to perform best, especially when few presence records are available^48^, while it is also the least affected by location errors in occurrences^49^. Maxent was run with the following modeling rules: (1) for species with 5–10 collection records linear features were applied, (2) for species with 10–14 records quadratic features were applied, while (3) for species with >15 records hinge features were applied^23^.

As a measure of the accuracy of the SDMs, we used the threshold independent and prevalence insensitive area under the curve (AUC) of the receiver operating characteristic (ROC) plot produced by Maxent. All measures of SDM accuracy require absences^50^. When these are lacking, as is the case here, they are replaced by pseudo-absences or sites randomly selected at localities where no species presence was recorded^20^. However, when SDM accuracy measures are based on presence-only data and pseudo-absences, the standard measures of accuracy (e.g. the often used measure AUC >0.7) do not apply^23^,^34^. Therefore, we applied the bias corrected null-model developed by Raes and ter Steege (2007) to test the AUC value of an SDM developed with all presence records against the AUC values expected by chance. However, this assumes that collection localities represent a random subset of the study areas environmental space. In many cases this is not a valid assumption due to collecting biases^51^,^52^.

To check for collecting bias in our dataset we tested whether our 3,068 collection localities were random subsamples of the environmental predictor space. To do this we divided each of the 16 environmental predictors into 10 equal-interval bins based on the ranges observed for China (34,230 grid cells)^53^. We then tested whether the observed frequency distributions represented by the 3,068 collection localities differed from those observed for whole China using a Chi-square test. This showed that for 14 of the 15 environmental predictors, our collection locations represented non-random subsamples of China’s environmental predictor space. To correct for this bias we developed a bias corrected null model by testing each species model AUC value against 1,000 AUC values that were generated randomly by subsampling from all the available collection localities only. When the observed AUC value fell in the top 95% of randomly generated AUC values, it was considered to have a significant non-random distribution and was used in our further analyses. For all the 6,828 available species of China, 6,560 species showed a significantly non-random distribution (AUC value ≥ 95% C.I.).

### Species diversity patterns and species turnover index

To define whether a species is present or absent in a grid cell, we need to select a threshold for Maxent prediction values. For species represented by 5–9 records we used either the ‘sensitivity specificity equality’ or the ‘sum maximization’ threshold, For SDMs represented by ≥10 records we used the fixed ‘10 percentile presence’ threshold. Once the threshold is set, a series of presence/absence layers of all the species becomes available. In the next step, a presence/absence matrix was created, the rows representing the 34,230 grid cells covering China and the columns representing the presence of the 6560 modeled species. Species richness was then mapped in ArcGIS 9.3.

In order to explain the species diversity patterns, we performed variation partitioning analysis. This method is especially useful when two sets (environmental and spatial predictors) of independent explanatory variables are involved in explaining the variation of an ecologically dependent variable. The nine terms of the trend-surface analysis regression equation were used to describe the spatial pattern in current species richness^54^:


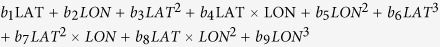


where LON refers to longitude and LAT refers to latitude. Environmental predictors were formed by contemporary climate predictors and soil predictors. The explanatory power (R_adj_^2^) of groups of variables (Environmental predictors, spatial predictors, combined predictors) was tested using the free software ‘spatial Analysis in Macroecology’ (SAM)^55^. Additionally, we used the same software to obtain the Moran’s I values to test for spatial autocorrelation (RSA) in model residuals. RSA may result from spatially structured biological processes, which violates the statistical independence of observations and may inflate the type I errors^56^. Influential environmental predictors for botanical richness patterns were identified by performing stepwise multiple regressions.

In order to identify phytogeographical regions we applied cluster analysis. For this the pair wise floristic distance between grid cell assemblages need to be measured using an appropriate metric. The most frequently used methods to measure the floristic distance between grid cell assemblages includes Sørensen/Bray–Curtis, Jaccard and Kulczynski, but these are all strongly affected by differences in species richness. This means that a change in community composition has a greater relative influence in relatively species-poor than in diverse assemblages, and if there is a large difference in richness between grid cells, the obtained values from these indices will also be large. Kreft and Jetz^11^ argued that for the purpose of biogeographical regionalizations richness-independent turnover is more informative, and therefore suggest the use of metrics that are least affected by the variation in richness. Therefore we used the beta-sim index based on distribution data at genus and species level:


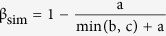


where a = the number of species shared between two grid cells and b and c represent the number of species unique to each grid cell. The 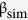 value ranges from 0 to 1, where 0 means pairs of grid cells have identical taxa lists, and 1 means that they share no taxa.

### Bioregionalization based on species compositional dissimilarity

We used the UPGMA method, which is frequently favoured and proven to perform among the best in floristic cluster analysis^11^,^57^. Determining the optimum number of final cluster groups is a long standing issue in biogeographical applications^45^. The indicator species analysis^58^ calculates the indicator taxa at each level of bifurcation in the cluster dendrogram and determines how well they fit the clusters (based on *p* values)^59^. The significance of the assignment to a cluster group is determined with a Monte-Carlo test using 1000 randomizations. For the cluster analysis, we used summed p-values for 2–30 cluster groups to detect the optimum level of grouping. We randomly drew 1000 grid cells for each analysis and repeated the procedure five times. We found that the lowest summed p-value was reached at eleven clusters, so we used this as the optimum level of grouping. We then performed the cluster analysis on the complete dissimilarity matrix, setting the cluster group level as 11. The indicator species analysis was performed using PCORD 5.0.

### Ordination and relative environmental turnover (RET)

Ordination is a widely used technique to produce low dimensional projections of multivariate data by arranging objects (in our case grid cell assemblages) along reduced axes based on taxonomic composition. We performed a NMDS ordination at species level, using the ‘metaMDS’ function of the vegan library^60^ in the statistical software R^61^. Pairwise distances were calculated using 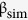. One hundred random start points were used to find a stable solution and to avoid local minima^11^. The stress value reflects the amount of error in the correlation between pairwise distances in the original 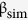 data matrix and a data matrix calculated from the NMDS plot, with 0 representing no error and 1 indicating a complete lack of correlation.

Once we got the NMDS results, the relative environmental turnover (RET) method was used to investigate the relationship between environmental predictors and the floristic regions of species turnover^62^. RET contains two types of analyses, one that matches the NMDS results with the environmental variables and a second that is based on the grid cell Getis-Ord Gi* hotspot statistic. First, all environmental variables included in species distribution modeling were re-sampled into 20 × 20 arc-minute and an environmental variable matrix was created. Secondly, we fitted NMDS results with the uncorrelated environmental variable matrix using the vector fitting of the *envfit* function from the vegan package in R statistical software. Thirdly, environmental variables that were significantly related to the patterns of turnover (P < 0.001, 999 permutations) were mapped onto the NMDS ordination plot as vectors.

In the final step, the Getis-Ord Gi* hotspot statistic was used to assess whether the environmental values within each phytogeographical region were significantly different from those of whole continental China^63^. Here, the clusters with Gi* values >2 or <−2 represent sets of cells that have environmental values significantly different from expected. The environmental layer processing and Getis-Ord Gi* hotspot statistic were performed in ARC-MAP 9.3.

## Additional Information

**How to cite this article**: Zhang, M.-G. *et al.* Using species distribution modeling to delineate the botanical richness patterns and phytogeographical regions of China. *Sci. Rep.* **6**, 22400; doi: 10.1038/srep22400 (2016).

## Supplementary Material

Supplementary Information

## Figures and Tables

**Table 1:** Result of stepwise multiple regressions between current species richness and environmental predictors.

Environmental predictors			
Pred.	R_adj_^2^	Beta	t
Annual Precipitation	0.641	0.451	85.527
Temperature Annual Range	0.706	−0.671	−53.584
Cation Exchange Capacity subsoil	0.746	−0.291	−54.353
Cation Exchange Capacity topsoil	0.776	0.269	60.386
Annual Mean Temperature	0.783	0.269	41.454
Isothermality	0.791	0.154	29.985
Soil drainage class	0.798	−0.091	−24.624
Base saturation topsoil	0.799	−0.065	−12.113
Rainfall Seasonality	0.801	−0.039	−10.315

Pred. (P < 0.05): the significant predictors that were included in the regression equations; R_adj_^2^: cumulative amount of explained variation for addition of each variable in the model; beta: standardized coefficient; t: t-value for the full regression model.

**Table 2:** Gi* statistic results for the grid cells comprising the 11 woody plants floristic regions of China.

	1	2	3	4	5	6	7	8	9	10	11
Annual Mean Temperature	**−3.37**	**−3.53**	**−3.45**	**−4.05**	**2.01**	**−3.63**	**2.75**	**−3.24**	1.08	**4.57**	**6.39**
Isothermality	**−3.54**	**−3.75**	**2.13**	**−2.66**	**−3.01**	**−2.73**	**−2.87**	**5.22**	****6.81****	**−2.99**	**3.25**
Temperature Annual Range	**5.10**	−0.80	**2.44**	**2.77**	**3.17**	**3.59**	1.42	**−3.29**	**−5.34**	**−3.81**	**−5.92**
Annual Precipitation	−1.55	−1.92	**−3.20**	**−3.14**	**−3.53**	**−2.41**	−0.42	−0.51	**2.32**	**5.67**	**7.29**
Precipitation Seasonality	**3.10**	−1.60	1.40	**−4.71**	**−2.65**	**2.78**	**2.17**	**3.87**	**3.23**	**−5.28**	**−3.63**
Base saturation% topsoil	**2.21**	**−3.66**	1.94	**3.44**	**−3.01**	**2.04**	1.86	**−2.27**	−0.93	−0.63	**−3.64**
CEC soil subsoil	**2.89**	**2.58**	−0.46	**3.17**	**−3.23**	**2.59**	1.24	**3.03**	1.20	**2.64**	−1.89
C:N ratio class topsoil	−1.35	**2.99**	−0.06	−0.06	**−2.65**	**−2.55**	**−2.40**	**2.37**	**4.21**	**2.76**	1.53
Effective soil depth	−0.14	**2.99**	**−2.37**	**−2.79**	**2.66**	**2.26**	1.23	**3.00**	**−2.44**	**2.62**	1.57
Soil drainage class	**2.91**	**−2.84**	**−2.94**	**−3.74**	1.27	**−2.7**	**2.66**	**−3.08**	**−3.92**	**3.68**	**2.85**

Black bolded means statistically significant (α = 0.05).
