# Supplementary material for: Using species distribution modeling to delineate the botanical richness patterns and phytogeographical regions of China
Source: Sci Rep. 2016 Mar 1;6:22400. doi: 10.1038/srep22400 (PMC4772162; doi:10.1038/srep22400)
Supplement: Supplementary Information [file srep22400-s1.pdf]

Using species distribution modeling to delineate the botanical richness patterns and phytogeographical regions of China

Ming-Gang Zhang [1, 2], J. W. Ferry Slik [3], Ke-Ping Ma [1*]

a. State Key Laboratory of Vegetation and Environmental Change, Institute of Botany, Chinese Academy of Sciences, Beijing 100093, China

b. Institute of Loess Plateau, Shanxi University, Taiyuan 030006, China

c. Faculty of Science, Universiti Brunei Darussalam, Jln Tungku Link, Gadong BE1410, Brunei Darussalam

* Correspondence and requests for materials should be addressed to K.P.M (kpma@ibcas.ac.cn)

Table S1. Spearman's rank correlation coefficients (r) for elevation and 5 bio-climate predictors, Bio01: Annual Mean Temperature. Bio03: Isothermality (P2/P7) (* 100) (P2: Mean Diurnal Rang). Bio07: Temperature Annual Range (P5-P6). Bio12: Annual Precipitation. Bio15: Precipitation Seasonality (Coefficient of Variation).

	Elevation	Bio01	Bio03	Bio07	Bio12
Bio01	-.638				
Bio03	.716	-.144			
Bio07	-.168	-.412	-.504		
Bio12	-.455	.514	-.147	-.610	
Bio15	.296	-.568	.179	.370	-.387

Table S2. Spearman's rank correlation coefficients (r) for the 9 soil predictors, BS_T: base saturation% topsoil. CE-S: CEC soil subsoil. CN-T: C:N ratio class topsoil. CP-T: organic carbon_pool. Depth: effective soil depth. Drain: soil drainagr class. NN-T: nitrogen% topsoil. Prod: soil production index. Text.: Textural class sub soil. CEC: cation exchange capacity.

	BS-T	CE-S	CN-T	CP-T	Depth	Drain	NN-T	Prod
CE-S	.483							
CN-T	-0.010	.272						
CP-T	.086	.430	.431					
Depth	.160	-.130	-.041	.061				
Drain	.446	.293	.022	.302	.442			
NN-T	.288	.540	.350	.591	-.175	.179		
Prod	.519	.180	-.231	.157	.420	.611	.090	
Text.	-.107	.065	.318	.289	-.028	-0.002	.233	-.106

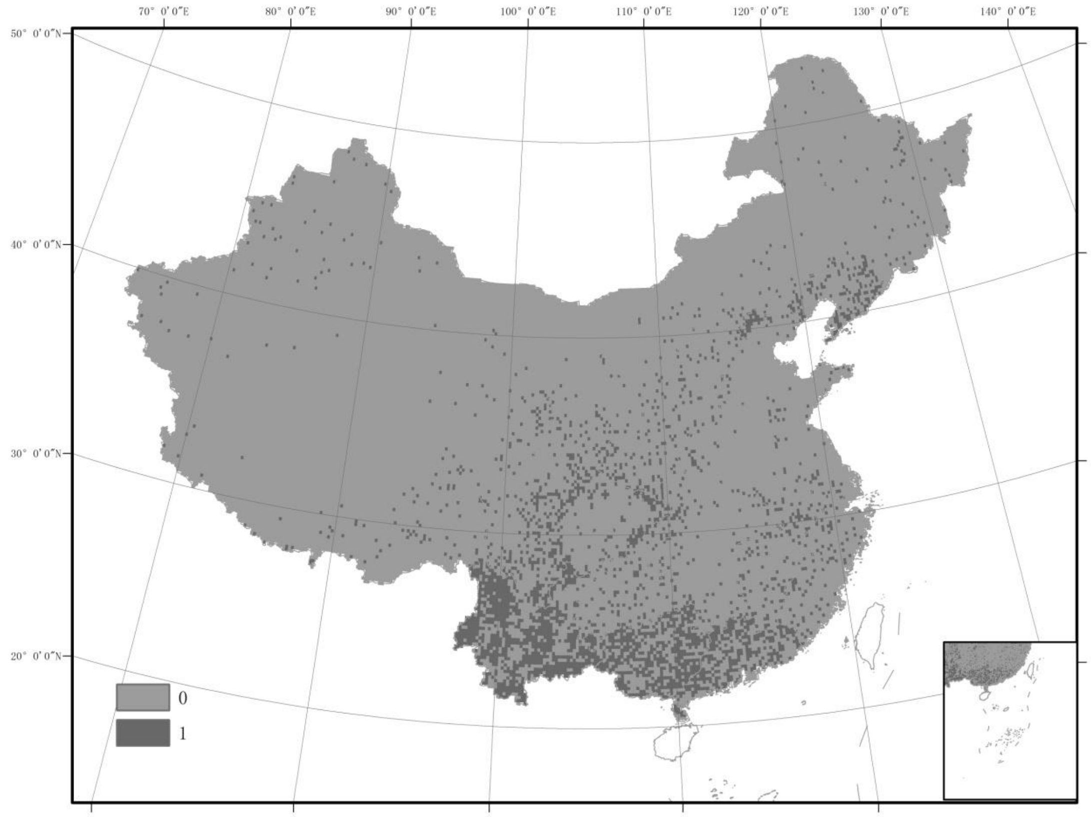

Figure S1. Grid cells that have been visited in the past (deep gray). Albers projection.

The map was plotted using ArcGIS 9.3 (http://www.esri.com/).

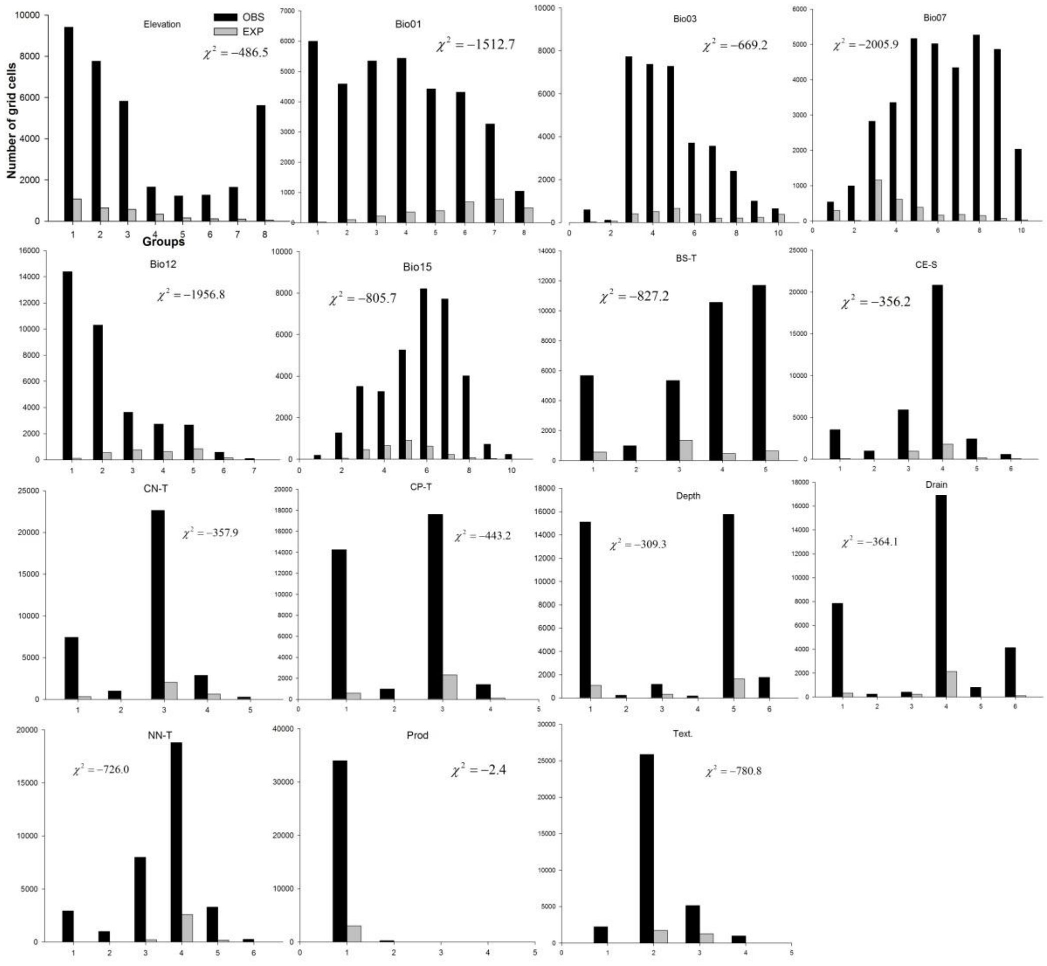

Figure S2. Chi square test for observed (deep gray histogram) and expected (light gray histogram) number of visited grid cells in 10 equal intervals of each predictor.

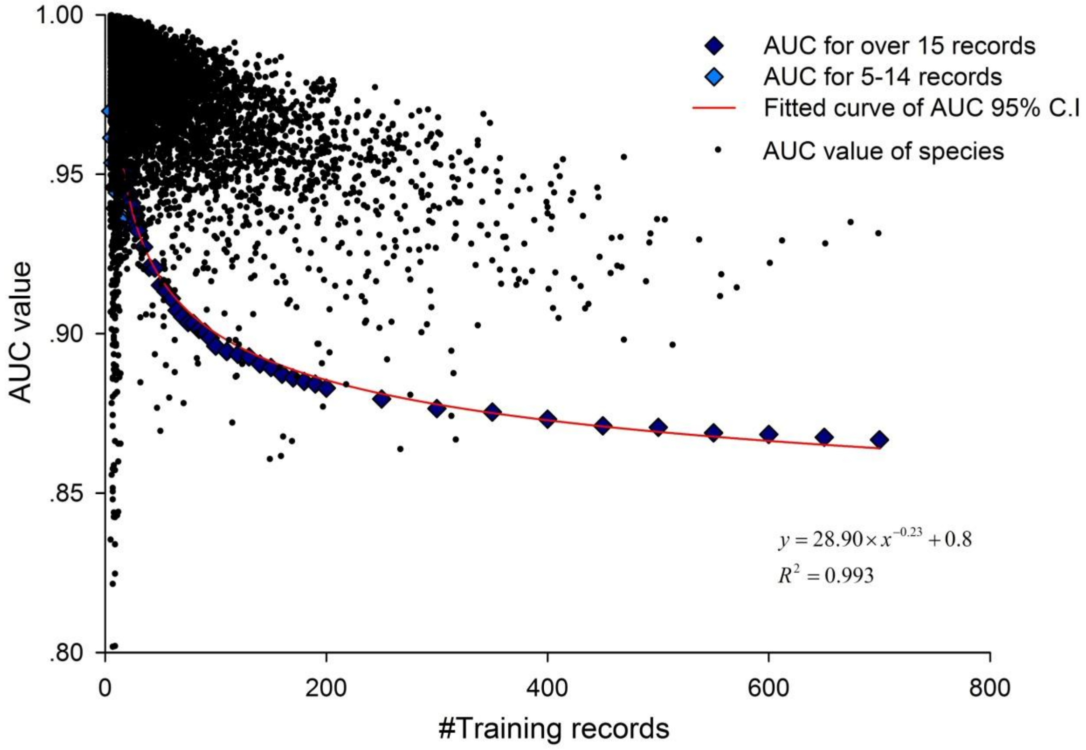

Figure S3. Species' distribution model (SDM) AUC values (round black dots) and the 95% C.I. AUC values of the environmentally bias corrected null-models (blue diamond). Fitted 95% C.I. AUC values for series of null-models connected by the red line, SDM AUC values that are higher than their corresponding 95% C.I. AUC value of the fitted null-model, significantly deviate from what would be expected by random.

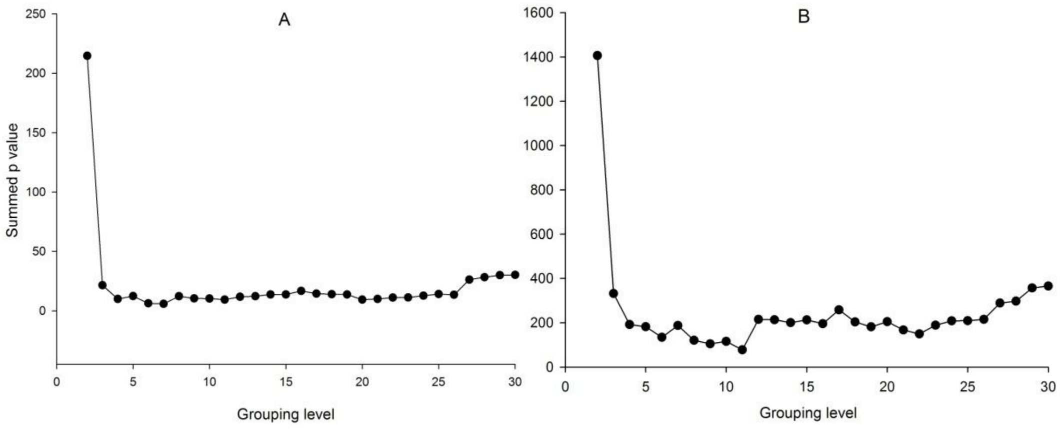

Figure S4. Summed p values in indicator species analysis and Monte-Carlo test. A: genus level; B: species level chance.

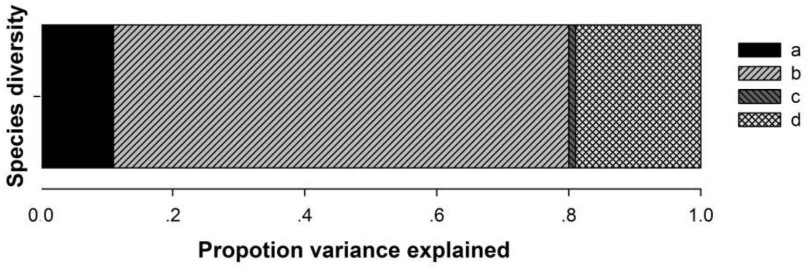

Figure S5. Result of variation partitioning of the stepwise multiple regressions for species richness in each grid cell, a= variance explained by environmental predictors, b= variance explained by environmental predictors combined with spatial predictors, c= variance explained by spatial predictors, d= residual variance.

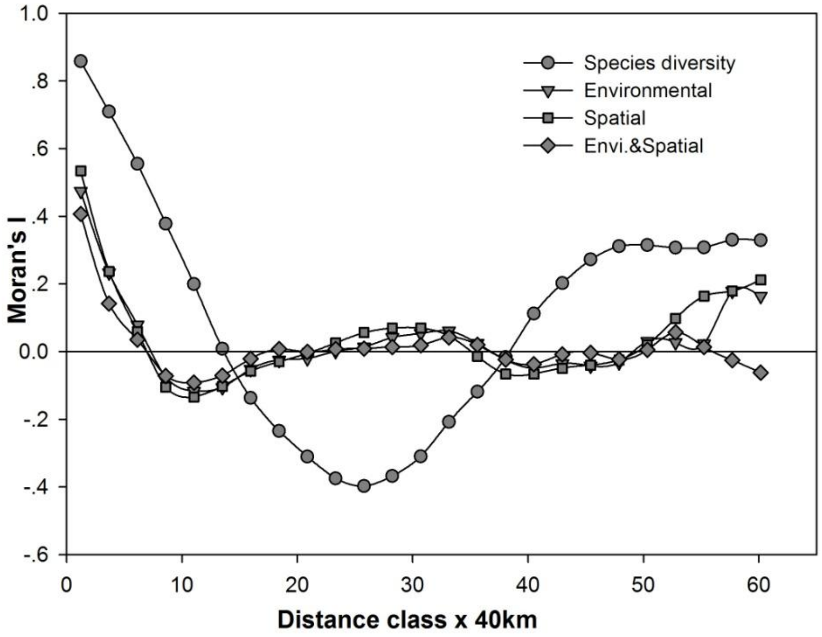

Figure S6. Moran's I value of current species diversity. Moran's I value for the residuals of linear regression results with environmental predictors (triangles), spatial predictors (squares) and environmental predictors combined with spatial predictors (diamonds).

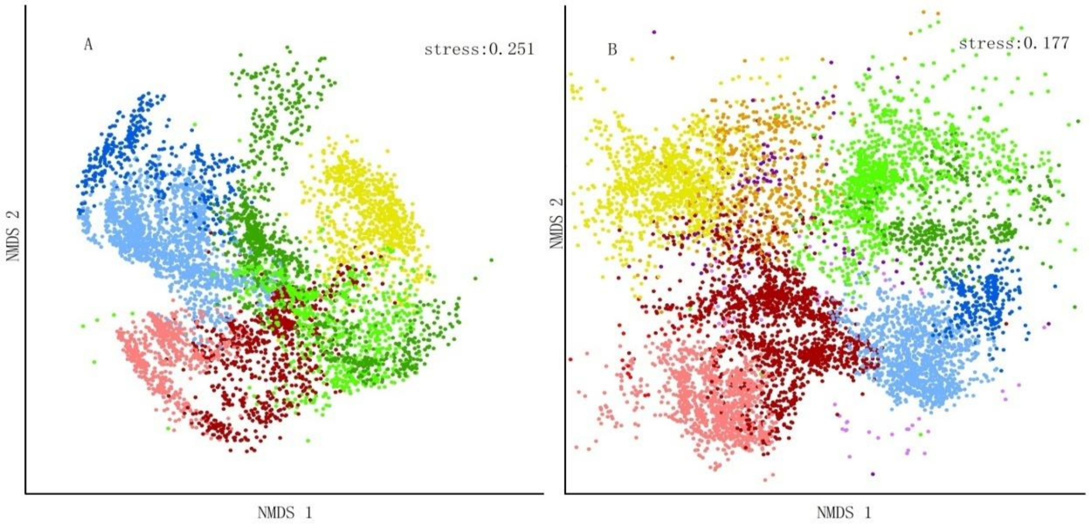

Figure S7. Biogeographical patterns of woody species inferred from non-metric multidimensional scaling (NMDS) ordination. The colors indicate the classification results in figure 2. A: genus level; B: species level.
